# The Effect of Low Dose OnabotulinumtoxinA on Cervical Dystonia in Hypermobile Ehlers-Danlos Syndrome

**DOI:** 10.5334/tohm.647

**Published:** 2021-10-25

**Authors:** Tina J. Wang, Antonio Stecco, Khashayar Dashtipour

**Affiliations:** 1 Loma Linda University School of Medicine, Department of Physical Medicine & Rehabilitation, US; 2 New York University School of Medicine, Department of Rehabilitation, US; 3 Loma Linda University School of Medicine, Department of Neurology, US

**Keywords:** OnabotulinumtoxinA, myofascial, fascia, sonoelastography, Ehlers-Danlos Syndrome, hypermobility, cervical dystonia

## Abstract

**Background:**

Many patients with hypermobile Ehlers-Danlos Syndrome (EDS) suffer from cervical dystonia. Intramuscular injection of botulinum toxin may exacerbate myeloradiculopathy or atlantoaxial subluxation in this patient population.

**Case:**

Three patients with hypermobile EDS underwent low-dose OnabotulinumtoxinA injections for cervical dystonia into myofascial sites selected using Fascial Manipulation diagnostic sequencing technique. All patients improved in clinical symptoms without complications.

**Results:**

Patients clinically improved on the TWSTRS by 16 points with demonstrated changes in deep fascia thickness decrease of 0.28 mm.

**Discussion:**

Low-dose OnabotulinumtoxinA injections into carefully selected sites is a safe and effective treatment in hypermobile EDS patients suffering from cervical dystonia.

## Introduction

Approximately 75% of the Ehlers-Danlos Syndrome (EDS) population suffers from cervical dystonia [1,2], a chronic neurological disorder associated with involuntary co-contraction of agonist and antagonist muscles which causes abnormal posture or movement of head and neck [3,4]. The majority of patients with cervical dystonia suffer from neck pain. These patients can suffer with lower quality of life if their symptoms are managed poorly [5].

Chemodenervation with botulinum toxins is generally accepted as the first-line therapy for cervical dystonia [6]. Different botulinum toxin serotypes have been shown in double blind placebo controlled clinical trials to be effective in cervical dystonia [5,7].

Cervical dystonia is particularly problematic and difficult to treat in the hypermobile population and can exacerbate myeloradiculopathy or atlantoaxial subluxation [8,9]. A significantly smaller dose of botulinum toxins may be warranted and sufficient in this patient population to avoid destabilization [10].

In this case series, low dose OnabotulinumtoxinA into selective myofascial sequences was used to effectively treat cervical dystonia in hypermobile EDS.

## Methods

Patients who had been clinically diagnosed with hypermobile EDS [11] and suffered from cervical dystonia (diagnosed by a board-certified Neurologist subspecialized in Movement Disorders) were identified in clinic. The initial visit for patients included in this case series occurred between March 25 2019 and July 15 2020. Data were extracted from medical records by the authors and the confidentiality of patient identity was maintained.

All patients had undergone physical therapy and prolotherapy [12,13] for craniocervical stabilization prior to OnabotulinumtoxinA injection. The study was exempted by IRB on 3/15/21; all patients were provided with general informed consent per IRB protocol.

The American Academy of Neurology (AAN) considers the classification of evidence as Class IV and classification of recommendation as U for a case series [14].

## Case Series Presentation

We present a series of 3 patients (≥18 years) with hypermobile EDS (independently meeting the 2017 diagnostic criteria [11]) who underwent low-dose OnabotulinumtoxinA injections for cervical dystonia.

Case Study 1. A 28-years-old right-handed female with hypermobile EDS diagnosed in 2020 was referred for management of myofascial neck and head pain. At the time of diagnosis, she was treated with manual therapy, neuropathic agents, topical cannabinoid, prolotherapy, and low dose naltrexone with limited benefit. The prominent posture of her CD was left torticollis and right laterocollis. The initial Toronto Western Spasmodic Torticollis Rating Scale (TWSTRS) [15] was 56.25. 100 U of OnabotulinumtoxinA were injected into multiple muscles (***Table 1***). One-month post injection TWSTRS was 36.25.

**Table 1:** TWSTRS, SCM deep fascia thickness, and SCM deep fascia stiffness before and after OnabotulinumtoxinA injection.


	PRE- INJECTION	POST- INJECTION	SITES OF INJECTION

Average			

TWSTRS	52 ± 4	36 ± 8	

Torticollis Severity	21 ± 5	4 ± 3	

Disability Scale	17 ± 8	1 ± 4	

Pain Scale	16 ± 2	12 ± 5	

SCM Deep Fascia Thickness	1.7 ± 0.5 mm	1.2 ± 0.2 mm	

Stiffness Deep Fascia to SCM Ratio	1.10 ± 0.38	1.33 ± 0.48	

Case 1			R semispinalis capitus 10 units × 2

TWSTRS	56.25	36.25	Fascial Plane Horizontal Intrarotation/Extrarotation:

Torticollis Severity	21	7	L SCM near mastoid process 10 units

Disability Scale	17	11	L SCM near clavicle 10 units

Pain Scale	18.25	18.25	L Splenius Capitus near Transverse process of C2–4 10 units × 2

SCM Deep Fascia Thickness	1.4 mm	1.1 mm	R Levator scapula 10 units × 2

Stiffness Deep Fascia to SCM Ratio	0.86	0.97	R Rhomboid minor at medial spine of scapula 10 units × 2

Case 2			R semispinalis capitus 10 units × 2

TWSTRS	49	15	Fascial Plane Diagonal Spiral Antemedial/retrolateral:

Torticollis Severity	26	3	R/L alar nasalis 4 units × 2

Disability Scale	18	3	L pectoralis minor at rib 2–4 4 units

Pain Scale	15	9	L SCM near sternoclavicular joint 10 units

SCM Deep Fascia Thickness	1.7 mm	1.4 mm	R Splenius capitus near C2–3 10 units × 2

Stiffness Deep Fascia to SCM Ratio	1.33	1.46	R upper trapezius border 10 units × 2

Case 3			R semispinalis capitus 10 units × 2

TWSTRS	50	20.5	Fascial Plane Horizontal Intrarotation/Extrarotation:

Torticollis Severity	17	2	L Auricularis anterior 4 units

Disability Scale	17	9	L SCM near mastoid process 10 units

Pain Scale	16	9.5	L SCM near clavicle 10 units

SCM Deep Fascia Thickness	1.9 mm	1.2 mm	L Splenius Capitus near Transverse process of C2–4 10 units × 2

Stiffness Deep Fascia to SCM Ratio	1.12	1.56	L Levator scapula 10 units L Rhomboid minor at medial spine of scapula 10 units × 2


Case Study 2. A 34-years-old right-handed male with hypermobile EDS diagnosed in 2019 was referred for management of neck and arm “pulling” and associated headaches. At the time of diagnosis, he was treated with prolotherapy, physical therapy, manual therapy, cranial osteopathy but because of partial control of recurrent symptoms, OnabotulinumtoxinA was pursued for the management of associated cervical dystonia. At examination his neck abnormal posture was right torticollis with the initial TWSTRS 49. Based on the examination 82 U OnabotulinumtoxinA were injected into different locations (***Table 1***); 1-month post injection TWSTRS was 15.

Case Study 3. A 49-years-old right-handed female with hypermobile EDS diagnosed in 2019 was referred for management of neck pain and headaches. Prior to diagnosis, she was treated unsuccessfully with radiofrequency ablation, trigger point injections, chiropractic care, and physical therapy. At the time of diagnosis, the patient was treated with prolotherapy, cyclobenzaprine, cannabis, and manual therapy with partial control of symptoms. The prominent posture of her cervical dystonia was left torticollis and right laterocollis. The initial TWSTRS was 50; 94 U of OnabotulinumtoxinA were injected into multiple muscles (***Table 1***). One-month post injection TWSTRS was 29.5.

Initial myofascial points were selected by a Fascia specialized Physical Medicine & Rehabilitation physician using a specific assessment methodology—Fascial Manipulation [16]—involving clinical examination by movement and palpatory verifications of specific points termed Centers of Coordination (CCs) and Centers of Fusion (CFs). A CC corresponded to the convergence of vectorial forces, into the deep fascia, generated by mono and biarticular motor units moving a joint in a specific direction. A CF corresponded to the converge of multiple vectorial forces, of intermediate directions between the three planes of space, into intermuscular septa or retinaculum. Palpation evaluation of these points included patient pain rate, radiation and the presence of tissue stiffness [16]. Dysfunctional segments were identified based on palpation evaluation and a hypothesis-driven differential by clinical history.

The patients then underwent a standard cervical dystonia assessment for head and neck position, localization of pain, muscle hypertrophy [6] with a Movement Disorder specialized Neurologist. For rotational excursion, the contralateral sternocleidomastoid and ipsilateral splenius capitus were selected as sites for OnabotulinumtoxinA injection. For laterocollis excursion/tilt, the ipsilateral levator scapulae, splenius capitus and semispinalis capitus were selected. For shoulder elevation, the ipsilateral trapezius and levator scapulae were selected.

**Figure 1:**
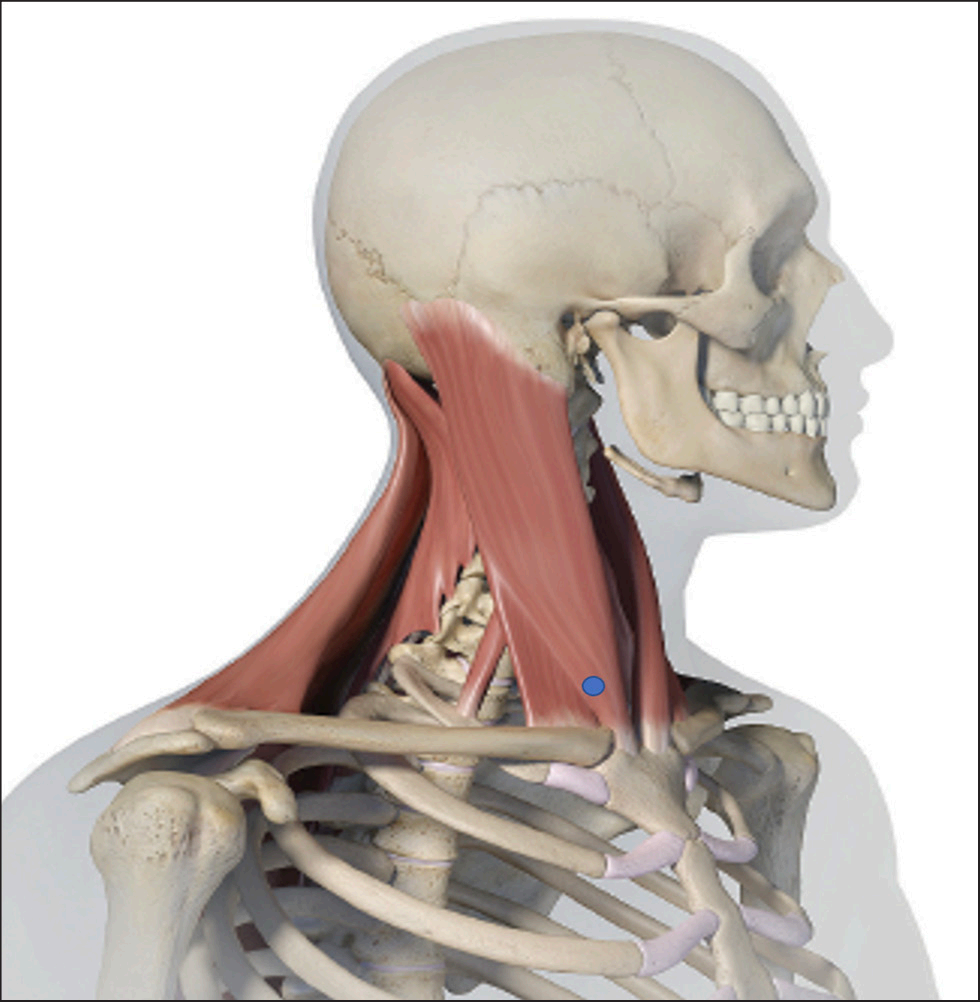
The right sternocleidomastoid was selected for injection under standard cervical dystonia assessment. This corresponded with the right CF (blue dot) located at the insertion site of the sternocleidomastoid at the sternoclavicular joint- allowing for more specificity in injection site selection.

The CCs/CFs selected for injection and belonging to the selected dysfunctional segments were compared to the muscles localized by standard dystonia assessment. All CC’s and CF’s corresponded with muscles identified by standard dystonia assessment with CC’s and CF’s allowing for more specificity for site of injection along the muscles identified by standard assessment. For instance, the CF right antemedial column was identified by fascial sequencing for injection and corresponded to the ipsilateral sternocleidomastoid selected by standard assessment for left rotation. Identification of the CF right antemedial column allowed for more specificity in injection site selection- at the insertion site of the sternocleidomastoid at the sternoclavicular joint. (***Figure 1***) In addition, CC’s and CF’s on the trunk were selected for injection to provide fascial balance and fell outside typical muscles selected for standard cervical dystonia assessment. Only semispinalis capitus selected via standard dystonia assessment fell outside of the sequenced fascial segments but were along the medial and retromedial fascial planes which are often manipulated for equilibrium disturbances.

The OnabotulinumtoxinA was reconstituted to 100 Units/1 mL with preservative-free 0.9% Sodium Chloride Injection, USP. An average of 5–8 CC’s and CF’s were injected with 4–20 units using a ¼ inch 30 gauge sterile needle for a total of 100 U, 82 U and 94 U per case per patient. The patients were followed-up at 4–8 weeks. Results are reported in ***Table 1***.

## Discussion

On average, the subjects had 16 points improvement in the TWSTRS greater than the 8 points for minimally important clinical change [15] without adverse effects reported in prior EDS patients [8,9,10]. Torticollis severity and disability scale had the largest average change, and pain scale had the least amount of change. This is consistent with functional improvement that is often clinically seen in hypermobile EDS patients with often minimal changes in pain level.

**Figure 2:**
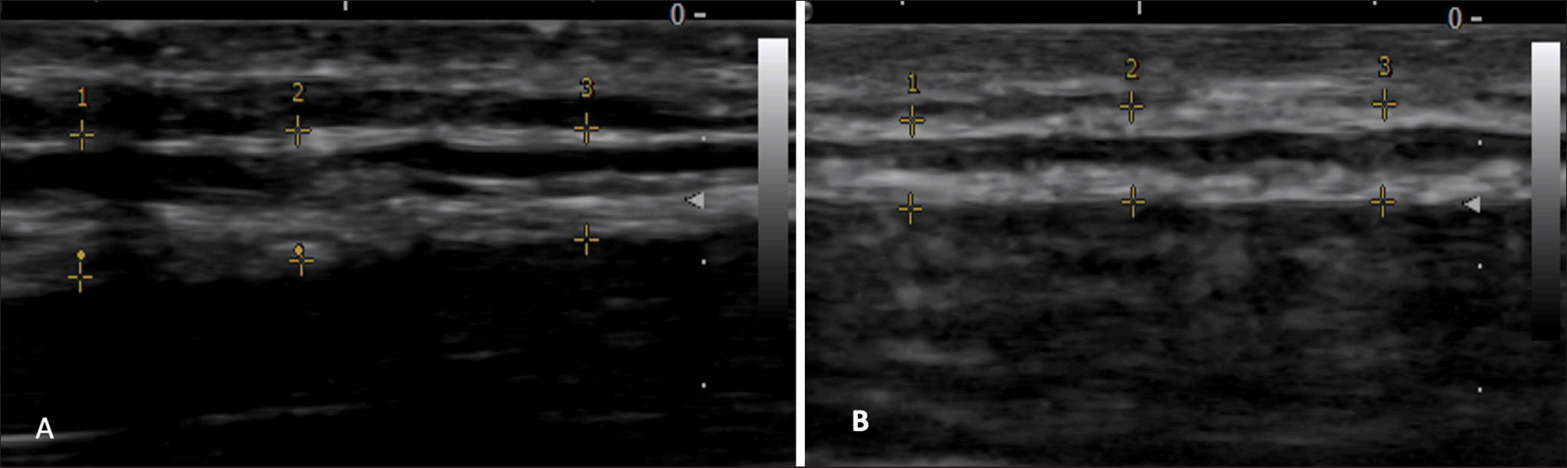
Changes in deep fascia thickness before ONABOTULINUMTOXINA injection **(A)** and after ONABOTULINUMTOXINA injection **(B)** showing decrease in thickness.

Many of the standard dystonia sites identified by physical examination of head rotation and tilt corresponded to identified dysfunctional fascial sequences. Low dose OnabotulinumtoxinA injections into CCs/CFs may aid in injection site specificity of selected muscles. OnabotulinumtoxinA injections may influence the fascial system with possible changes in fascial properties like thickness (***Figure 2***). Total dose used in these patients were less than standard dosing but no less effective, possibly secondary to the close relationship of some CCs/CFs to the location of endplates in the selected muscles [17].

The limitation of this study was the case study nature. It would be interesting to validate our findings in larger patient groups across healthcare settings and operators. Additional studies with more than one cycle of OnabotulinumtoxinA injection may help to further validate effects. Nevertheless, despite the preliminary nature of our data, this study highlights important characteristics in the hypermobile EDS population.
